# Adverse Events Following Yellow Fever Vaccination in Older Adults in Colombia, 2024–2025: A National Pharmacovigilance Analysis

**DOI:** 10.3390/vaccines14090757

**Published:** 2026-08-30

**Authors:** Heriberto Vásquez-Serna, Carlos Eduardo Jiménez-Canizales, Carlos Alexander Huertas-Caro, Diana Urrego-Ricaurte, Johan Estiven Vargas-Vargas, Luis Gabriel Forero Gutiérrez, Laura Rodríguez, Olga Corredor, Gina Hurtado, Lysien I. Zambrano, Alfonso J. Rodríguez-Morales

**Affiliations:** 1 Directorate of Epidemiology and Demography, Ministry of Health and Social Protection, Bogotá 110311, Colombia; hvasquez@minsalud.gov.co (H.V.-S.); cjimenezc@minsalud.gov.co (C.E.J.-C.); chuertas@minsalud.gov.co (C.A.H.-C.); durregor@minsalud.gov.co (D.U.-R.); 2 Grupo de Investigación ENDOCARDIO +, Universidad del Tolima, Ibagué 730006299, Colombia; jevargas@fucsalud.edu.co; 3 Deputy Directorate of Operations Management, Ministry of Health and Social Protection, Bogotá 110311, Colombia; lgforero@minsalud.gov.co (L.G.F.G.); lrodrigueza@minsalud.gov.co (L.R.); ocorredor@minsalud.gov.co (O.C.); ghurtadog@minsalud.gov.co (G.H.); 4 Faculty of Health Sciences, Universidad Tecnológica Centroamericana (UNITEC), Tegucigalpa 11101, Honduras; lysien.zambrano@unah.edu.hn; 5 Faculty of Health Sciences, Universidad Científica del Sur, Lima 15307, Peru; 6 Grupo de Investigación Biomedicina, Faculty of Medicine, Fundación Universitaria Autónoma de las Américas-Institución Universitaria Visión de las Américas, Pereira 660003, Colombia

**Keywords:** yellow fever, vaccination, post-vaccination adverse events, elderly, epidemiological surveillance, Colombia

## Abstract

**Introduction:** Yellow fever re-emerged as a major public health threat in the Americas during 2024–2025, with increasing numbers of cases and deaths, predominantly among unvaccinated individuals. Although the live-attenuated 17D yellow fever vaccine is highly effective, adults aged ≥60 years have an increased risk of serious adverse events following immunization (AEFIs). We assessed the safety profile of yellow fever vaccination in Colombia, with particular emphasis on older adults, and contextualized the observed risks within an epidemic setting characterized by active transmission and high mortality. **Methods:** We conducted an observational, descriptive study using secondary data from Colombia’s Expanded Program on Immunization and the national VigiFlow pharmacovigilance system. Individuals vaccinated against yellow fever between 1 October 2024 and 31 July 2025 were included. We analyzed reported AEFIs by seriousness, sex, and age group. We calculated rates per 100,000 vaccinated individuals with 95% confidence intervals and compared them between adults aged ≥60 years and younger individuals. We further stratified older adults into five-year age groups. **Results:** Among 3,915,966 vaccinated individuals, 425 AEFIs were reported in 233 unique cases. The overall AEFI reporting rate was 10.85 per 100,000 vaccinated individuals, and the serious AEFI reporting rate was 2.94 per 100,000. Adults aged ≥60 years had substantially higher overall AEFI reporting rates than those aged <60 years (54.05 versus 6.12 per 100,000, respectively), corresponding to a rate ratio of 8.8. Serious AEFI reporting rates were 11.90 and 1.96 per 100,000, respectively, corresponding to a rate ratio of 6.1. The highest serious AEFI reporting rate was observed among adults aged 80–84 years, at 49.82 per 100,000. Nine fatal outcomes were reported; following causality assessment, none was considered attributable to the vaccine. **Conclusions:** Reported AEFIs following yellow fever vaccination were more frequent among adults aged ≥60 years, with a particularly marked increase in the oldest age groups. Nevertheless, the absolute serious AEFI reporting rate remained low, at approximately 1.2 per 10,000 vaccinated older adults, and no reviewed fatal outcomes were attributed to vaccination. In settings of active yellow fever transmission, vaccination decisions for older adults should therefore be guided by individualized benefit–risk assessment rather than age alone, accompanied by careful screening, active pharmacovigilance, and clear risk communication. Vaccination remains the principal intervention for preventing yellow fever-related deaths.

## 1. Introduction

Yellow fever is an acute viral hemorrhagic disease [1], frequently manifesting with fever, myalgia, and jaundice [2]. A proportion of patients progress to a toxic phase, with liver and kidney failure, reaching a case fatality rate of approximately 40% [1,3]. Among patients who develop serious disease, approximately half die within 7 to 10 days [1,3].

Despite the availability of a safe and effective vaccine for more than eight decades, yellow fever has re-emerged as a threat to regional health security [4,5]. Between 2024 and 2025, an increase in cases was documented in different countries of South America [6], with reports occurring outside endemic areas such as the Amazon basin and a high case fatality rate [7]. Most confirmed cases occurred in unvaccinated individuals [8,9]. Therefore, the World Health Organization (WHO) classified the regional risk as “high” [3,10].

Colombia, an endemic country, experienced a marked increase in reported cases. According to the National Institute of Health (INS), 125 cases of yellow fever were identified in 2025, of which 51 were fatal, resulting in a case fatality rate of 40.8%. Eighty percent of the cases occurred in elderly males, and in this group, the case fatality rate increased to 65.5% [11,12,13,14,15]. It is considered that changes due to immunosenescence in advanced age, as well as chronic vascular inflammatory changes, represent a greater biological vulnerability associated with the potential for exaggerated vascular damage during infection, thus increasing the case fatality rate [16,17,18,19,20,21].

The live attenuated 17D virus vaccine is the most important, safe, and affordable preventive measure; it induces seroprotection in more than 95% of patients, with prolonged protection and 98.6% effectiveness in healthy adults after a single dose [22,23,24,25]. International recommendations indicate vaccinating the population over 9 months of age living in risk areas and travelers going to these areas, including those over 60 years of age without risk comorbidities, restricting vaccination of this population group only in specific cases [25,26,27].

Despite these recommendations and the growing evidence of vaccine safety, in many places the expected vaccination thresholds are not being reached, and lower adherence has been described in those over 60 years of age, precisely the age group with the highest mortality and, at the same time, with the highest risk of serious adverse events [28,29,30].

Given the live virus nature of the yellow fever vaccine, serious adverse events such as viscerotropic disease (YEL-AVD) and neurotropic disease (YEL-AND) have been reported. These events are rare and related to underlying conditions in vaccinated individuals. The risk of complications increases with age because immunosenescence impairs innate and adaptive immune responses in older adults [17,28,30,31]. Importantly, the WHO position paper notes that reported YEL-AVD and YEL-AND cases have occurred after primary yellow fever vaccination rather than booster immunization, an issue particularly relevant when interpreting safety estimates from mass vaccination campaigns [25,26,27].

In Colombia, the yellow fever vaccine is included in the Expanded Program on Immunization (EPI), is provided free of charge, and has historically been targeted by age and geographic risk. Before the current outbreak, vaccination was not universally compulsory, although it was strongly recommended for residents of and travelers to areas at risk [11,32]. In July 2024, cumulative vaccination coverage among persons aged 1–59 years was estimated at 64% nationally, with substantial heterogeneity by age: 93% among those aged 1–14 years, 34% among those aged 15–18 years, 71% among those aged 19–40 years, and 31% among those aged 41–59 years. As yellow fever cases emerged and spread geographically, the Ministry of Health progressively intensified its vaccination strategy. Circular 018 of October 2024 established vaccination from 9 months of age in municipalities within the affected endemic corridor, while subsequent regulations in early 2025 extended vaccination to adults aged ≥60 years in prioritized areas of Tolima. On 16 April 2025, Resolution 691 declared a national health emergency. It established a risk-stratified strategy: in high- and very-high-risk municipalities, it targeted all susceptible persons aged ≥9 months, including adults aged ≥60 years, with a coverage goal of at least 95%. The policy targeted susceptible individuals rather than requiring revaccination for those with documented prior yellow fever vaccination. Vaccination teams were instructed to verify prior vaccination through physical or digital vaccination cards, institutional records, or immunization information systems before administering the vaccine. The 2025 national guideline maintained a single-dose schedule; therefore, individuals with a verified previous dose were not routinely targeted for revaccination. When a previous vaccination could not be verified, however, vaccination was recommended unless a contraindication was present, while individuals could also declare a previous dose and decline revaccination [33]. Consequently, some doses administered during the emergency campaign may have been repeat vaccinations for people whose previous immunization was undocumented. The policy required health authorities to offer and implement vaccination rather than impose universal compulsory vaccination actively. However, it authorized mobility controls and verification of vaccination status in very-high-risk areas. By 28 July 2025, approximately 90% of the estimated susceptible population in very-high-risk municipalities had been vaccinated, compared with approximately 35% in high-risk municipalities, illustrating both the rapid scale-up of the campaign and the remaining heterogeneity in population protection [32].

Furthermore, the controversy between protection and safety has generated cautionary messages regarding higher-risk population groups, which may lead to vaccination restrictions among older adults, precisely the population with the highest mortality rate from the infection. This study aimed to estimate AEFI reporting rates following yellow fever vaccination in Colombia, with particular emphasis on adults aged ≥60 years, and to characterize age-related safety signals in the context of active yellow fever transmission.

## 2. Methods

### 2.1. Design and Population

This was an observational, descriptive study using secondary data, with an analytical component for disproportionality. All individuals vaccinated against yellow fever and registered in the Expanded Program on Immunization (EPI) between 1 October 2024 and 31 July 2025 were included, as well as cases reported as adverse events following immunization (AEFIs) to the national pharmacovigilance system via the VigiFlow platform administered by the National Institute for Food and Drug Surveillance (INVIMA). The analysis was stratified by age groups (9–23 months, 2–19 years, 20–59 years, and ≥60 years) and, within the older age group, by five-year age increments (60–64, 65–69, 70–74, 75–79, 80–84, and ≥85 years).

### 2.2. Sources of Information

We used official sources of individual vaccination records from the Expanded Program on Immunization (EPI) for yellow fever as the population denominator, and individual safety case reports (ICSRs) notified to the INVIMA national pharmacovigilance system and registered in VigiFlow (INVIMA) as the numerator. During the study period, the yellow fever vaccines supplied through the Colombian EPI included vaccines manufactured by Bio-Manguinhos/Fiocruz (Rio de Janeiro, Brazil) and STAMARIL, manufactured by Sanofi Pasteur (Lyon, France). The Bio-Manguinhos vaccine was used throughout the initial and expanded phases of the campaign, while STAMARIL was also added to the EPI supply in 2025. Records of serious AEFIs reviewed by the technical expert committee of the National Institute of Health were incorporated as a supplementary source for causality classification. The study investigators did not perform or select causality assessments. We included all completed causality classifications by the National Institute of Health technical expert committee that were available in the surveillance records at the time of data extraction. Consequently, cases without a completed or available causality assessment at the study cutoff were not included in the causality analysis. In accordance with the Colombian national AEFI assessment framework, the committee applied standardized Brighton Collaboration case definitions, when applicable, for clinical validation and causality assessment of reported events. For suspected YEL-AVD, the committee evaluated cases using the standardized Brighton Collaboration case definition for yellow fever vaccine-associated viscerotropic disease [34]. Reports with adverse events following immunization before vaccination or with negative intervals or intervals greater than 365 days, as well as records without seriousness classification, were excluded. Although individual EPI records could include information on previous yellow fever vaccination, the analytic dataset lacked sufficient completeness and consistency to distinguish primary from repeat vaccination reliably. Consequently, primary versus repeat vaccination status was not available as an analysis variable.

### 2.3. Variables and Instruments

The primary outcome variables were the rate of adverse events per 100,000 vaccinated individuals, both overall and stratified by seriousness, age group, sex, and month; the type of adverse event reported; and the interval between vaccination and the onset of the adverse event (in days). Exposure variables included age group, sex, campaign type, and the patient’s department of residence. For this analysis, we classified AEFIs as serious or non-serious according to the seriousness criteria used in the VigiFlow pharmacovigilance system. An AEFI was considered serious if it resulted in death, was life-threatening, required hospitalization or prolongation of existing hospitalization, resulted in permanent disability, or involved a congenital anomaly. AEFIs not meeting any of these criteria were classified as non-serious. This classification reflects the event’s seriousness based on its outcome and required medical intervention, rather than the clinical intensity of individual symptoms.

### 2.4. Statistical Analysis

We calculated AEFI rates as the ratio of observed events to administered doses in each group, expressed per 100,000 vaccinated individuals, with 95% confidence intervals estimated using an exact Poisson distribution.

For age disproportionality, we calculated the Reporting Odds Ratio (ROR) for each AEFI type to compare reporting frequencies across groups, with 95% confidence intervals estimated using the Woolf method. We defined a positive signal as a 95% CI with the lower bound >1.

For predictors of seriousness, we fitted a binary logistic regression model with seriousness classification (serious/not serious) as the dependent variable and age (in years) and sex as predictors. Adjusted odds ratios with their 95% confidence intervals and *p*-values are reported. Available individual-level variables for risk-factor assessment were limited primarily to age and sex. Information on comorbidities, frailty, immunosuppression, concomitant medications, previous yellow fever vaccination, and other potential clinical determinants was not systematically complete across pharmacovigilance reports. We therefore did not include these factors in the multivariable model.

The analytical approach aimed to detect and characterize pharmacovigilance signals rather than estimate causal vaccine-attributable incidence. Rates calculated using the vaccinated population as the denominator therefore represent AEFI reporting rates. We used the ROR to assess disproportionate reporting of specific AEFIs between groups, and it should not be interpreted as a relative risk or incidence ratio. Similarly, the logistic regression evaluated factors associated with seriousness among reported AEFIs and did not estimate the risk of developing an AEFI among all vaccinated individuals. All these analyses remain susceptible to spontaneous-reporting limitations, including under-reporting, stimulated reporting, differential reporting between population groups and periods, and incomplete clinical information.

Given the small number of reported events in the oldest five-year age strata, we additionally compared AEFI seriousness between adults aged ≥80 years and those aged 60–79 years using an ROR with 95% confidence intervals estimated by the Woolf method.

We did not perform a formal quantitative benefit–risk analysis. We used comparisons with yellow fever morbidity and mortality only to provide epidemiological and public health context for the observed pharmacovigilance signals. The findings should not be interpreted as direct comparisons between equivalent measures of vaccine-associated risk and disease risk.

We used contingency tables with chi-square tests to compare the distribution of AEFI by sex, age group, and campaign type. We performed all analyses in R version 4.4.1. A significance level of α = 0.05 was used.

### 2.5. Ethical Considerations

This study is a secondary analysis of anonymized, non-identifiable public health surveillance data. VigiFlow data were managed in accordance with the WHO’s VigiBase platform usage guidelines and current Colombian data protection regulations (Law 1581 of 2012). According to Resolution 8430 of 1993, issued by the Colombian Ministry of Health, this research is classified as low risk. The study was approved by an external ethics committee with approval code CIMO-IPS-CEI_EXT-002_2026 [32].

## 3. Results

Between October 2024 and July 2025, 3,915,966 people were vaccinated against yellow fever, and 425 adverse events following immunization (AEFIs) were reported, corresponding to 233 unique cases. The overall AEFI reporting rate was 10.85 per 100,000 vaccinated individuals (95% CI: 9.8–11.9), of which 115 (27.1%) met the seriousness criteria, representing a serious AEFI reporting rate of 2.94 per 100,000 vaccinated individuals (95% CI: 2.4–3.5). During the vaccination period, the first months of the campaign (October, November, and December 2024) saw sustained vaccination activity, with only two AEFI reports in VigiFlow, suggesting underreporting during the campaign’s expansion phase (Table 1).

**Figure 1:**
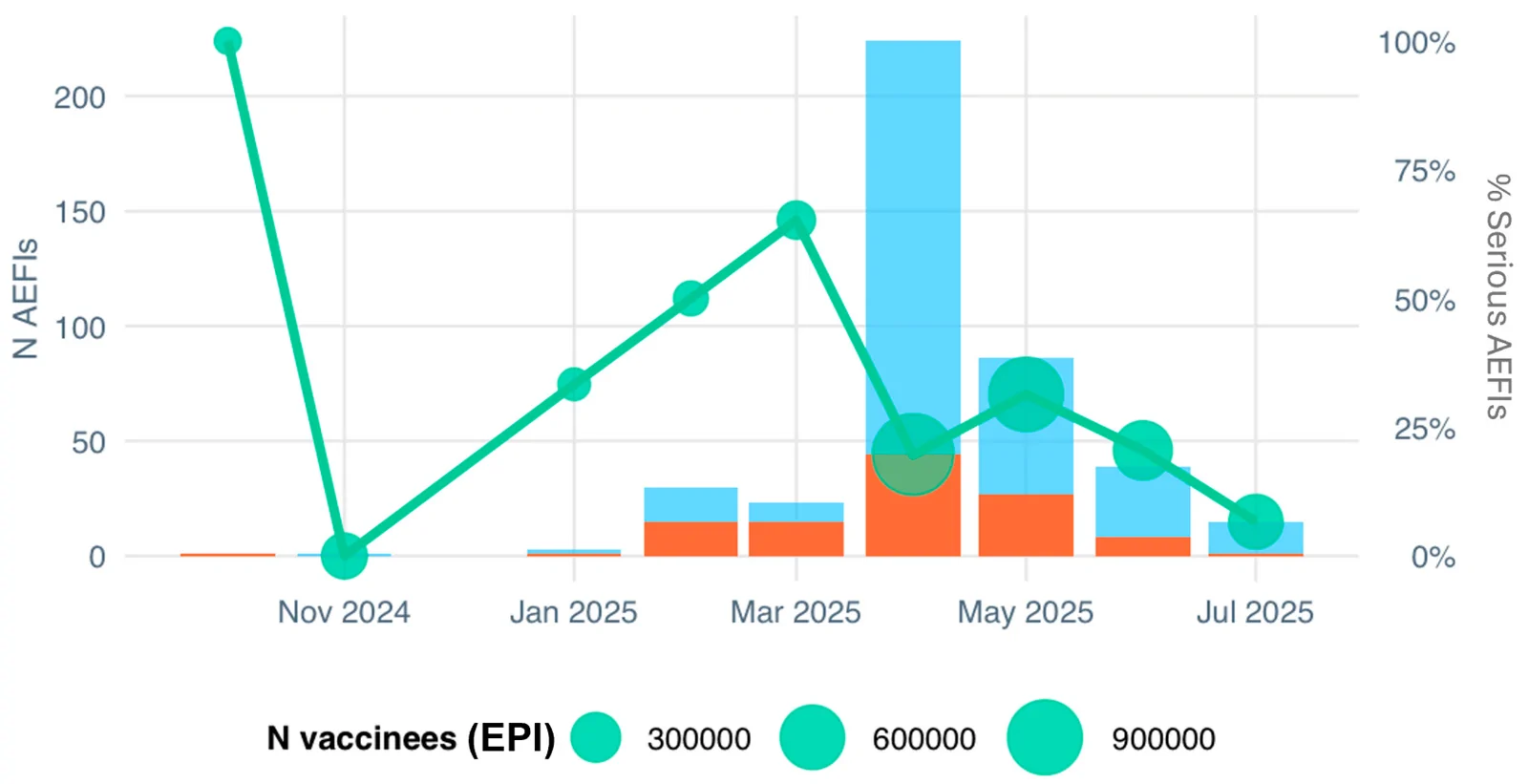
AEFIs per month: volume and proportion. Bars: total AEFIs (blue) and serious AEFIs (orange). Green line: percentage of serious AEFIs; dot size: number of vaccinated individuals recorded in the Expanded Program on Immunization (EPI). **AEFIs**: adverse events following immunization.

Reporting activity increased from January 2025 to March 2025, primarily representing serious AEFIs. Subsequently, April 2025 had the highest vaccination activity (1,129,442) and AEFI reporting (224), with a lower proportion of serious events, marking the start of mild-event reporting and correcting for selection bias. This trend continued to decline until July 2025 (Figure 1, Table 1).

**Figure 2:**
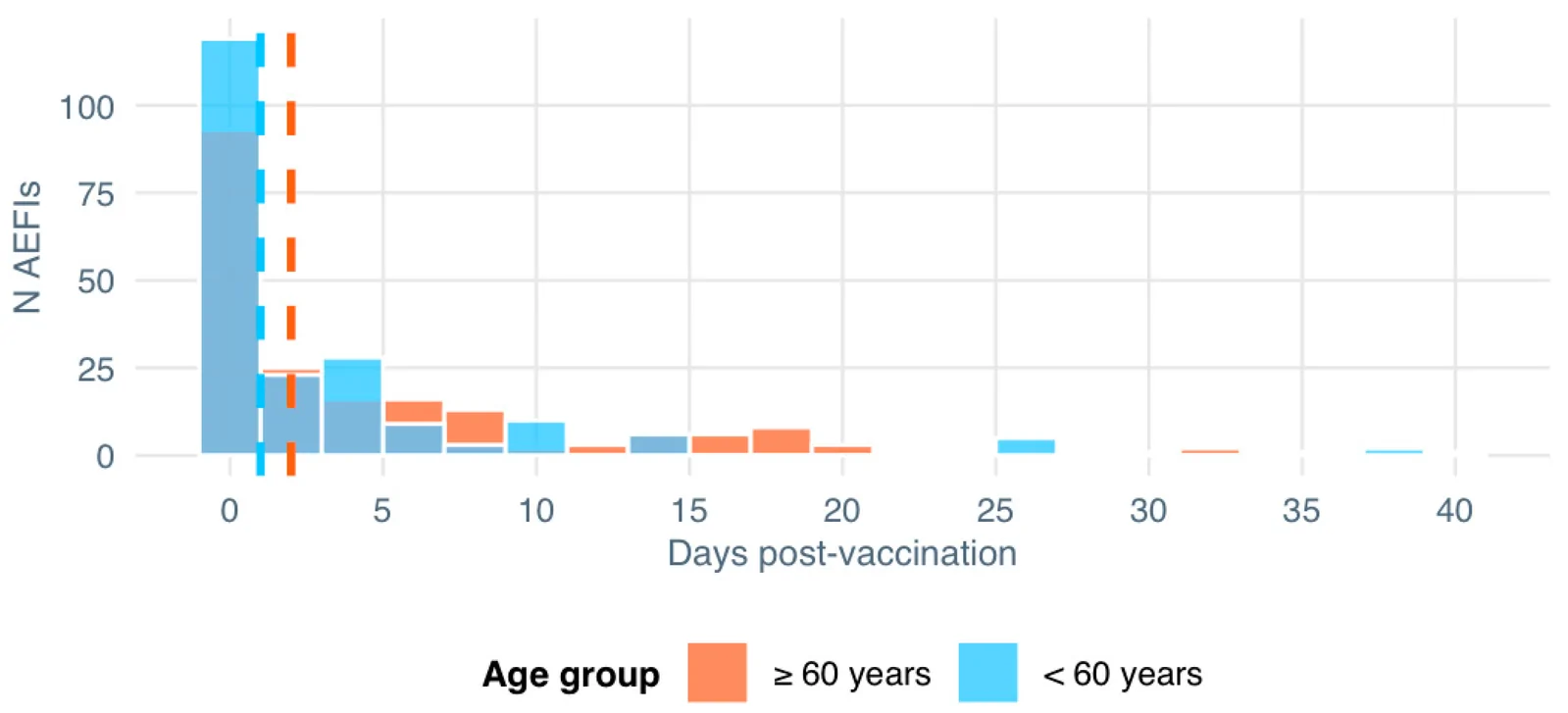
Distribution of the vaccination interval at the start of AEFIs. The dashed line indicates the median per group; negative values and those >365 days were excluded. **AEFIs:** adverse events following immunization.

Additionally, the temporal distribution of AEFIs showed a marked concentration in the first hours and days post-vaccination, consistent with the expected profile for a live attenuated virus vaccine, with 95.7% of events recorded within the first 30 days. When broken down by event type, non-serious AEFIs had a median onset of 1 day (IQR: 0–4), meaning half of them appeared in the first 24 h post-vaccination, while serious events had a median onset of 2 days (IQR: 0–11, *p* < 0.001), with a more dispersed distribution (Figure 2). This highlights that although most events occur within the first 48 h and correspond to expected acute reactions, potentially serious events are distributed over a wider window that does not always coincide with the period of greatest post-vaccination surveillance, leading to under-detection of more serious cases, particularly in older adults.

Regarding the distribution by sex, men accounted for 54.8% of reported AEFIs (233 events in 118 cases) while representing 45% of the vaccinated population, with an overall AEFI reporting rate of 13.23 per 100,000 vaccinated individuals (95% CI: 11.6–14.9), compared with 8.91 per 100,000 among women (95% CI: 7.7–10.2), corresponding to a rate ratio of 1.5 (Table 2). The serious AEFI reporting rate was 4.66 per 100,000 among men and 1.53 per 100,000 among women. Overall, 35.2% of AEFIs reported in men met the seriousness criteria, compared with 17.2% among women (*p* < 0.001) (Table 2).

**Figure 3:**
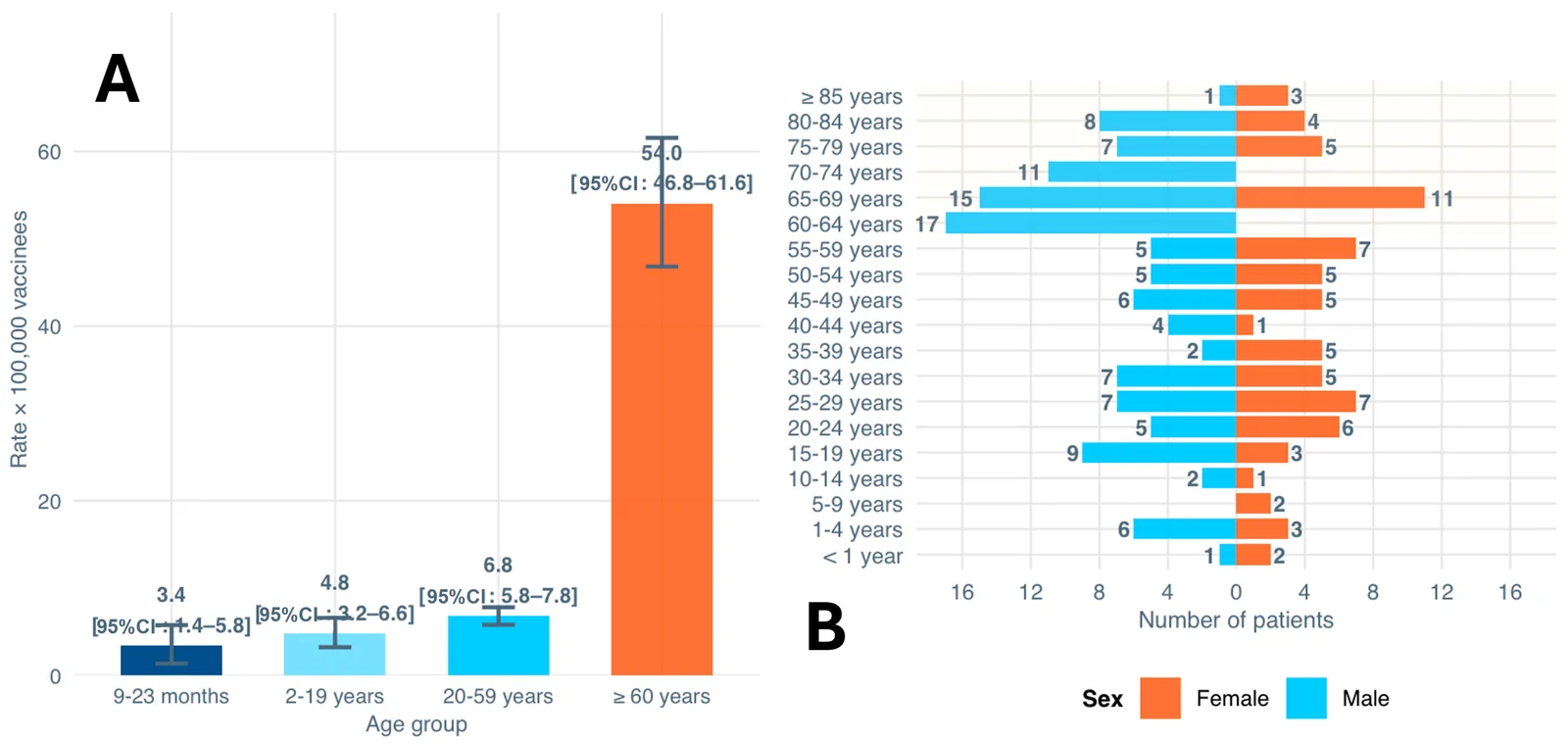
(**A**) AEFI rate by age group (×100,000 vaccinated), with 95% CI Poisson (denominator). (**B**) Population pyramid of patients with AEFIs following yellow fever vaccination; unique patients by five-year period and sex, Sep 2024–Jul 2025. **AEFIs**: adverse events following immunization.

When broken down by age groups, in those under 60 years of age, the AEFI reporting rates remained low and within a narrow range, with the 9–23 month group at 3.39 per 100,000 (95% CI: 1.4–5.8), the 2–19 year group at 4.83 (95% CI: 3.2–6.6), and the 20–59 year group at 6.78 (95% CI: 5.8–7.8). However, in the group over 60 years of age, the rate increased to 54.05 per 100,000 (95% CI: 46.8–61.5), representing a rate ratio of 8.8 compared to the population under 60 years of age and 8.0 compared to the previous group (20–59 years) (Figure 3A, Table 3). Similarly, when discriminating by sex, a higher number of reported AEFIs was observed in men over 60 years of age (Figure 3B).

**Figure 4:**
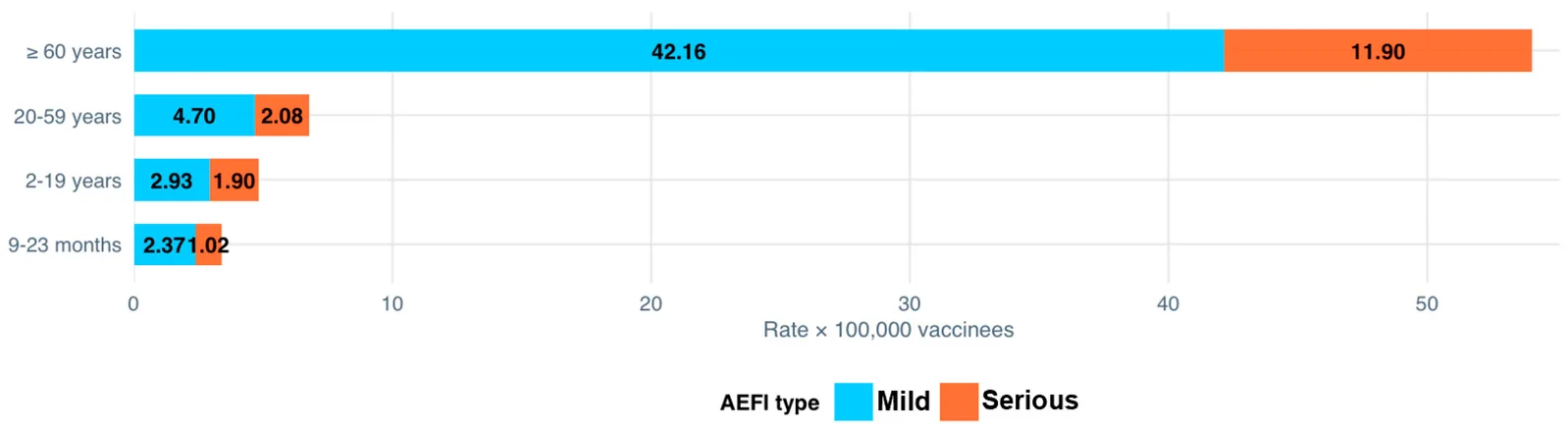
Serious and mild AEFI rates by age group (×100,000), breakdown by seriousness. **AEFIs**: adverse events following immunization.

When the age groups <60 years were pooled, the overall AEFI reporting rate was 6.12 per 100,000 vaccinated individuals, compared with 54.05 per 100,000 among adults aged ≥60 years, corresponding to a rate ratio of 8.8. Similarly, the serious AEFI reporting rate was 1.96 per 100,000 among individuals aged <60 years and 11.90 per 100,000 among those aged ≥60 years, corresponding to a rate ratio of 6.1 (Figure 4).

**Figure 5:**
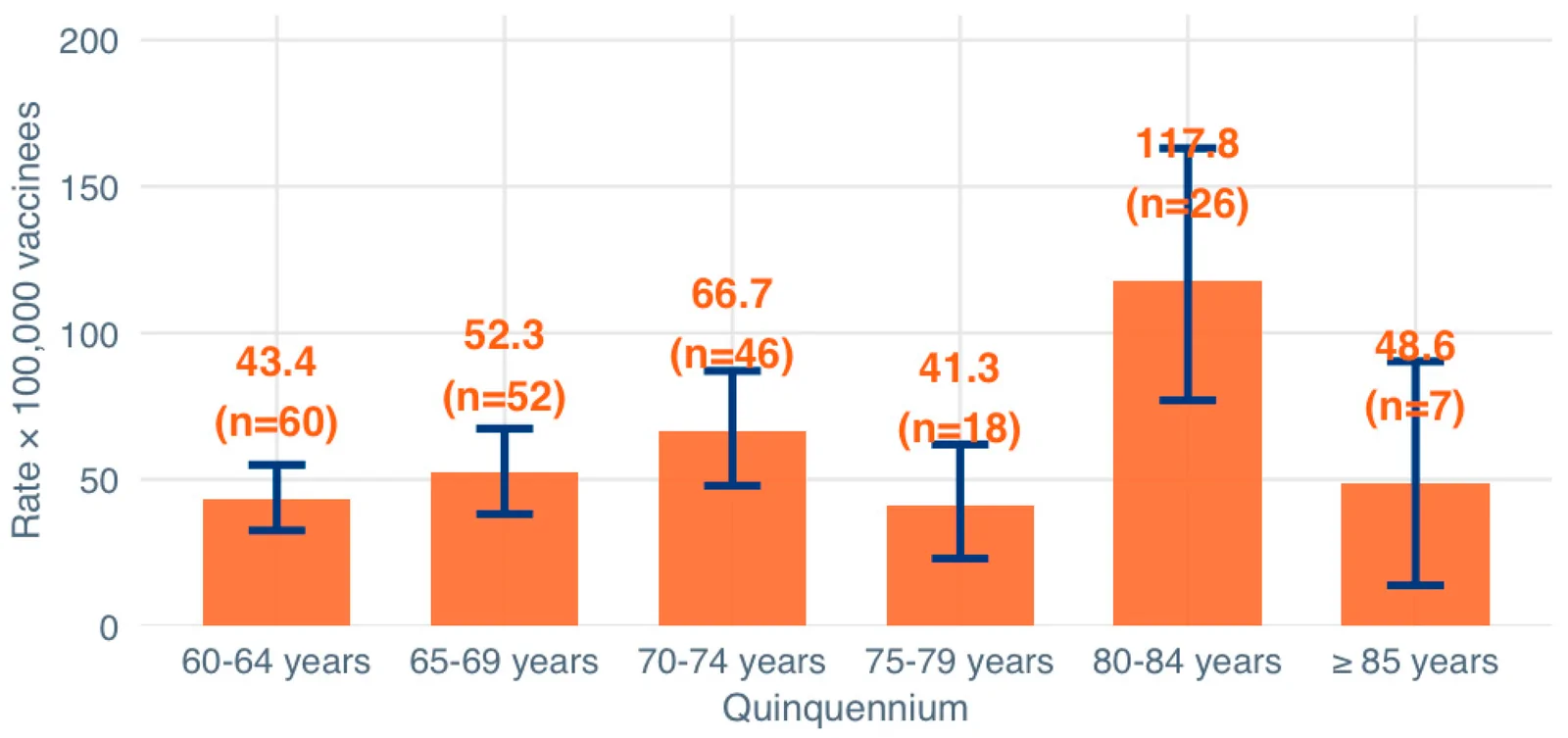
Rate of AEFIs per five-year period in people over 60 years of age per 100,000 vaccinated, adjusted by the number of doses administered; *95%CI Poisson*. **AEFIs**: adverse events following immunization.

Given the higher AEFI rate in the over-60 age group, we emphasized and disaggregated this group by five-year age ranges. Because reported AEFIs were small in some of the oldest five-year age strata, we also grouped adults aged ≥60 years into broader categories (60–69, 70–79, and ≥80 years) for a sensitivity analysis. The corresponding AEFI reporting rates were 47.1, 56.9, and 90.4 per 100,000 vaccinated individuals, respectively. Rate heterogeneity remained statistically significant across these broader age groups (Poisson regression likelihood-ratio test, *p* = 0.01), with the highest reporting rate observed among adults aged ≥80 years. Because some five-year strata among adults aged ≥60 years had relatively few AEFIs, we grouped these individuals into broader age categories (60–69, 70–79, and ≥80 years) for sensitivity analysis. We evaluated heterogeneity in AEFI reporting rates across these categories using Poisson regression, with the number of vaccinated individuals as the exposure offset. These findings indicate age-related heterogeneity in AEFI reporting but should not be interpreted as demonstrating statistically significant pairwise differences between all individual age categories. An AEFI reporting rate of 43.41 per 100,000 vaccinated individuals was observed in the 60–64-year age group (95% CI: 32.6–55.0), 52.28 in the 65–69 age group (95%CI: 38.2–67.4), 66.74 in the 70–74 age group (95%CI: 47.9–87.1), 41.30 in the 75–79 age group (95%CI: 22.9–62.0), 117.60 in the 80–84 age group (95%CI: 77.0–163.1), and 48.63 in the ≥85-year age group (95%CI: 13.9–90.3) (Figure 5). The lower rate in the 75–79 age group than in the other groups likely reflects the sample size (18 AEFI in 43,582 vaccinated individuals). The 80–84 age group had the highest rate in the entire cohort (including those under 60 years of age), at 2.7 times that of the 60–64 age group.

**Figure 6:**
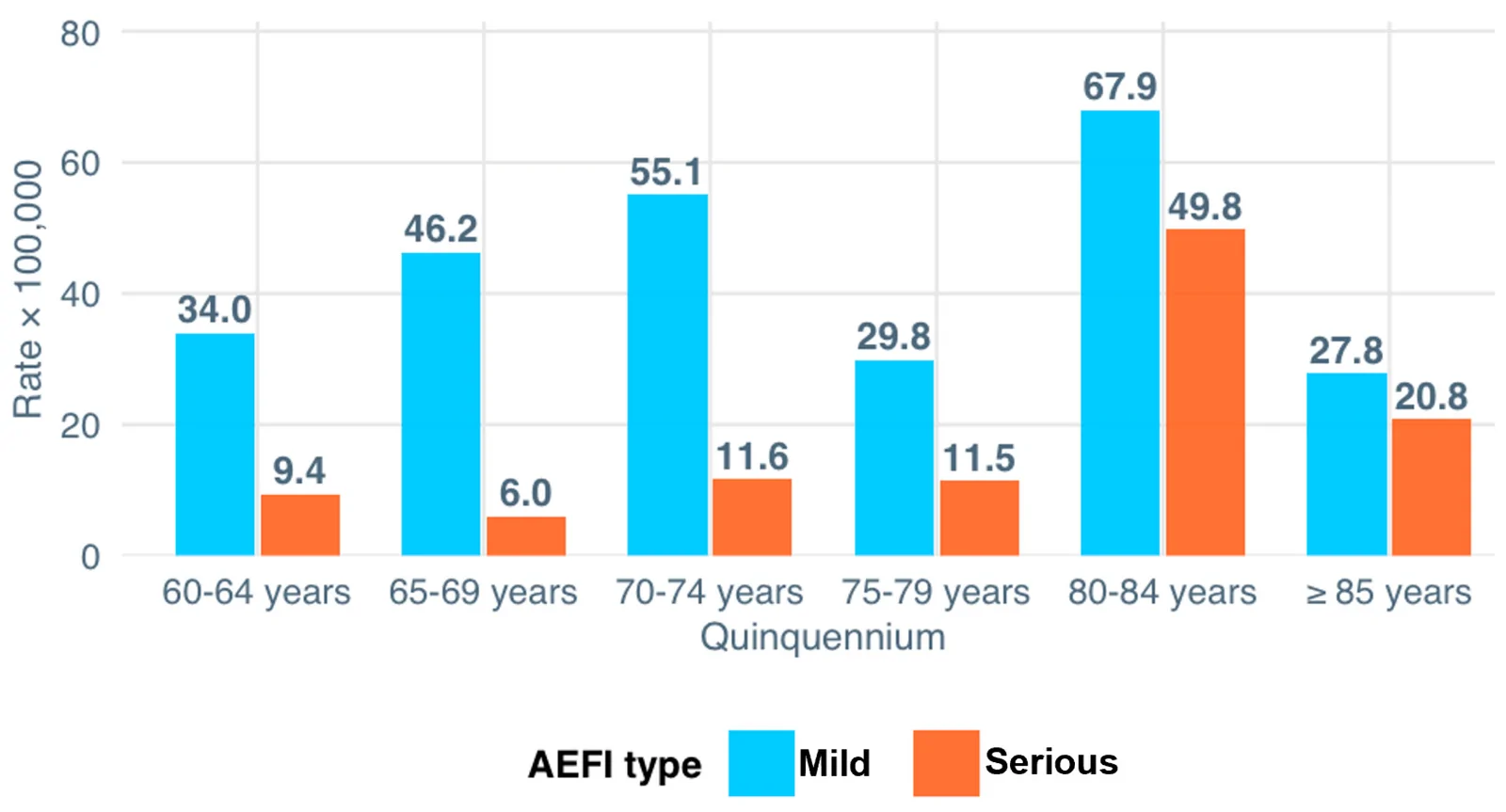
Rate of serious and mild AEFIs per five-year period in people over 60 years of age per 100,000 vaccinated, adjusted by the number of doses administered. **AEFIs**: adverse events following immunization.

When we examined serious AEFIs across five-year age strata, the rates did not show a monotonic age gradient. Rather, the principal increase was observed among adults aged ≥80 years. To provide a more stable comparison given the small numbers in the oldest five-year strata, we compared adults aged ≥80 years with those aged 60–79 years. Among reported AEFIs, 14 of 33 (42.4%) in adults aged ≥80 years were serious, compared with 32 of 176 (18.2%) among adults aged 60–79 years. The reporting odds ratio (ROR) for seriousness in adults aged ≥80 years versus those aged 60–79 years was 3.32 (95% CI: 1.51–7.30). Thus, serious AEFIs were disproportionately represented among reported events in adults aged ≥80 years. The individual five-year estimates are retained descriptively but should be interpreted cautiously because of the small number of events in the oldest strata (Figure 6).

**Figure 7:**
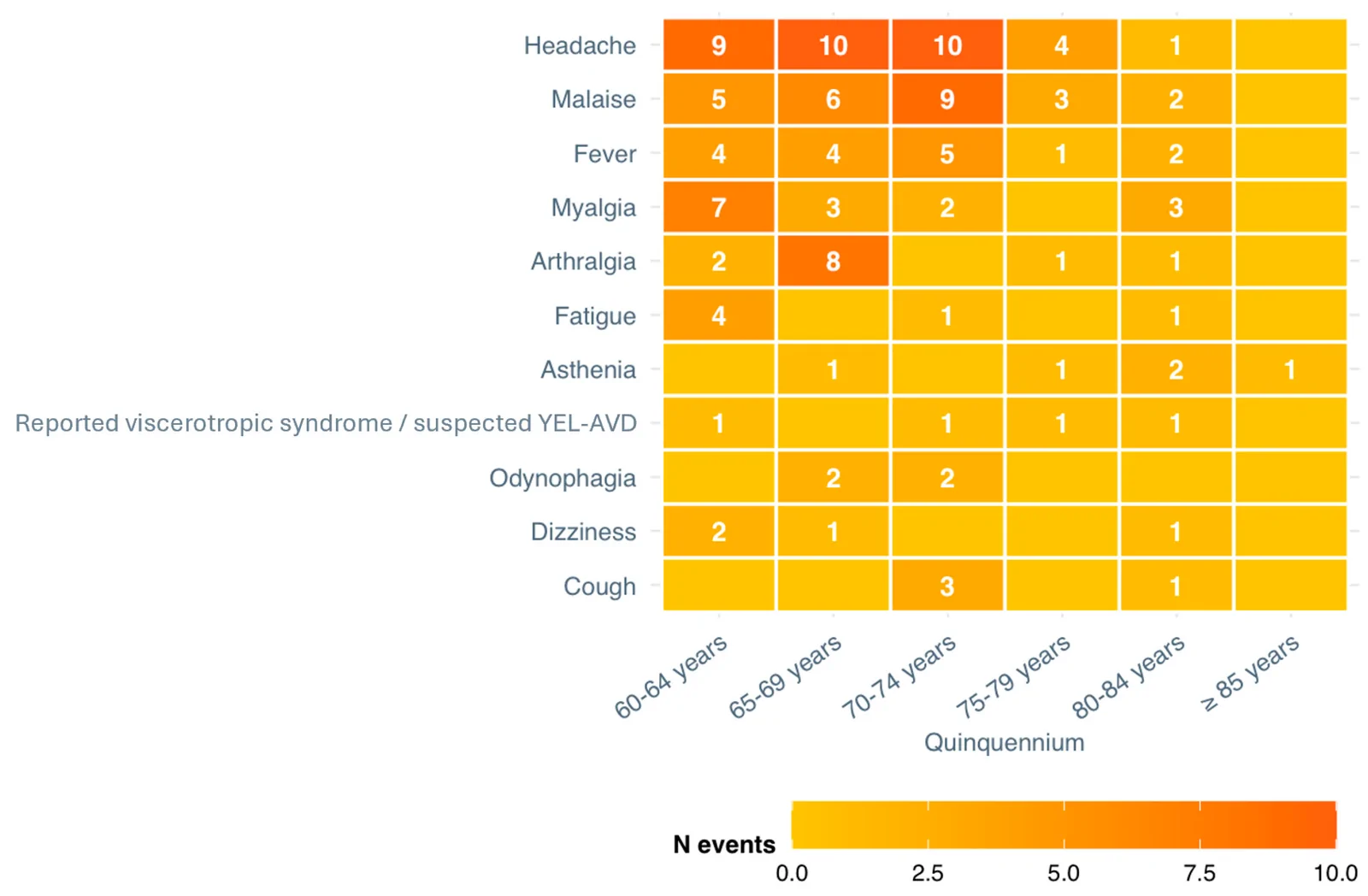
Absolute frequency of the main clinical manifestations classified as AEFIs per five-year period in those aged ≥60 years. **AEFIs**: adverse events following immunization.

When symptoms reported as AEFIs were presented over a 5-year period among those over 60 years of age, headache and general malaise were the most frequent manifestations in the 60 to 74 age group, followed by fever (Figure 7).

Four AEFI reports among adults aged ≥60 years were initially recorded with clinical presentations compatible with a viscerotropic syndrome or suspected YEL-AVD. These reports should not be interpreted as four confirmed cases of vaccine-associated viscerotropic disease. Two of the four reports occurred among fatal cases and underwent expert causality assessment; both were classified as coincidental and therefore did not meet the criteria for vaccine-attributable YEL-AVD. The remaining two reports were non-fatal and had no completed causality assessment available at the study cutoff and thus were classified as suspicious/probable.

**Figure 8:**
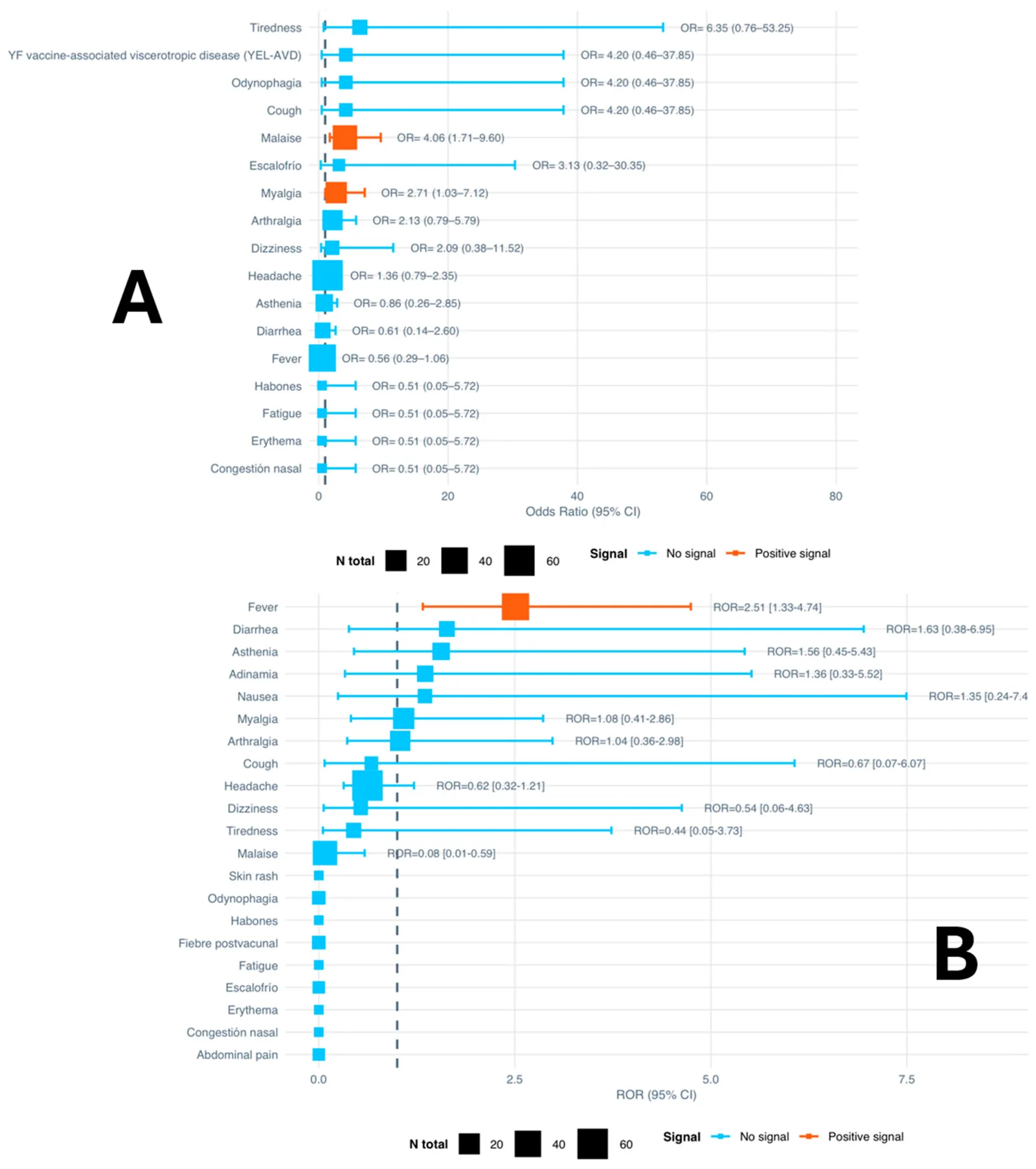
Disproportionality analyses of reported AEFIs following yellow fever vaccination: (**A**) Reporting odds ratios for individual AEFI types comparing adults aged ≥60 years with those aged <60 years. Values >1 indicate disproportionate reporting among older adults. Only AEFI types with ≥3 reports were included; a signal was considered positive when the lower bound of the 95% CI exceeded 1. (**B**) Reporting odds ratios for seriousness, comparing serious with non-serious reports for each AEFI type. Values >1 indicate disproportionate representation among serious AEFIs. The vertical reference line indicates an OR/ROR of 1.0. AEFIs, adverse events following immunization; OR, odds ratio; ROR, reporting odds ratio; CI, confidence interval. **AEFIs**: adverse events following immunization.

When performing the disproportionality analysis based on age and seriousness, in the comparison of adults aged ≥60 years versus those aged <60 years, two manifestations showed positive signals, general malaise (OR = 4.06; 95% CI: 1.71–9.60) and myalgia (OR = 2.71; 95% CI: 1.03–7.12) (Figure 8A); that is, when an AEFI occurs in someone over 60 years old, it is four times more likely to be general malaise and almost three times more likely to be myalgia than in a younger person.

Regarding seriousness, fever was the only reported AEFI showing a disproportionality signal (ROR = 2.51; 95% CI: 1.33–4.74), indicating that fever was disproportionately reported among serious compared with non-serious AEFIs (Figure 8B). This highlights the importance of fever as a clinical warning sign that should not be overlooked in vaccinated older adults.

Age, analyzed as a continuous variable, was not independently associated with seriousness at the conventional level of statistical significance (OR = 0.991; 95% CI: 0.981–1.000; *p* = 0.058), whereas male sex was independently associated with higher odds of a serious AEFI (OR = 2.589; 95% CI: 1.642–4.155; *p* < 0.001) (Table 4). Further adjustment for clinical characteristics was not possible because the pharmacovigilance database incompletely recorded comorbidities and other potential individual-level risk factors.

### 3.1. Classification of Causality and Characterization of the Deceased

Among serious AEFI reports, 20 individual cases had a completed causality classification available in the surveillance records at the time of data extraction. Investigators did not select these cases; rather, they represented all cases with a completed expert-committee causality assessment available at the study cutoff. However, the specific analysis of the 9 cases with a fatal outcome, based on the Individual Case Safety Reports (ICSRs) submitted to INVIMA, showed that none of the deaths were attributable to the vaccine: among the fatal cases with available causality assessment, none was considered attributable to the vaccine; assessed deaths were classified as coincidental and were explained by underlying comorbidities or intercurrent events and explained by underlying comorbidities or intercurrent events (acute myocardial infarction, sepsis, stroke, endocarditis, and respiratory distress). Two fatal reports were initially described as having clinical presentations compatible with a viscerotropic syndrome. However, after review by the National Institute of Health technical expert committee using the Brighton Collaboration case definition and the national causality-assessment framework, neither was considered vaccine-attributable YEL-AVD. The committee classified both events as coincidental because criteria for confirmation were not fulfilled. Thus, the term “viscerotropic syndrome” refers to the initially reported clinical presentation and should not be interpreted as a confirmed diagnosis of YEL-AVD. The most frequent comorbidities were hypertension, dyslipidemia, diabetes, chronic obstructive pulmonary disease, and neurodegenerative diseases. Among fatal cases with available product information, the implicated vaccines corresponded predominantly to Bio-Manguinhos/Fiocruz lots (23K0929, 24F0617, and 24F0618), with one report involving STAMARIL (Sanofi Pasteur^®^) (Table 5). Additionally, five serious cases with available temporal and clinical information had symptoms compatible with yellow fever before vaccination, supporting the subsequent coincidence classification. This finding emphasizes the importance of careful pre-vaccination clinical assessment to identify symptoms potentially related to an ongoing illness and to distinguish pre-existing disease from events occurring after immunization; it should not be interpreted as supporting additional restrictions on yellow fever vaccination beyond established contraindications and precautions.

### 3.2. Epidemiological Context of AEFI Reporting and Yellow Fever Mortality

Among adults aged ≥60 years, the serious AEFI reporting rate was 11.9 per 100,000 vaccinated individuals, corresponding to slightly more than one reported serious event per 10,000 vaccinees. Importantly, this figure reflects a pharmacovigilance reporting rate, not the incidence of causally vaccine-attributable serious adverse reactions. Moreover, none of the fatal outcomes with available causality assessment were attributed to vaccination. Yellow fever, in contrast, was associated with substantial mortality during the Colombian outbreak, particularly among older and unvaccinated individuals. These measures should not be interpreted as directly comparable estimates of risk because they have different denominators and derive from different surveillance processes. Accordingly, the present study does not estimate a vaccine-attributable risk–benefit ratio, number needed to vaccinate, or number needed to harm. Instead, we present the disease mortality data to contextualize the pharmacovigilance findings within the epidemiological circumstances under which vaccination decisions were made. Nevertheless, the coexistence of active viral transmission, high disease seriousness, and relatively infrequent reporting of serious post-vaccination events provides relevant epidemiological context for individualized vaccination decisions. These findings support current recommendations that vaccination of older adults should be based on assessment of exposure risk, contraindications, and individual clinical characteristics rather than chronological age alone.

### 3.3. Data Quality

During the analysis, limitations in data quality were identified that condition the interpretation: heterogeneity in the nominal registration of the PAIweb, underreporting, missing data in clinical variables in the pharmacovigilance reports (age, batch, comorbidities, and causality classification), and the absence of a complete causality classification for all serious AEFIs.

## 4. Discussion

The overall pharmacovigilance profile observed in this study was predominantly characterized by non-serious, acute post-vaccination events. Of the 425 reported AEFIs, 310 (72.9%) were classified as non-serious. Their temporal pattern was also predominantly acute: non-serious AEFIs had a median onset of 1 day (IQR: 0–4), and most reported events occurred within the first 48 h after vaccination, consistent with the expected short-term reactogenicity profile of yellow fever vaccination. Nevertheless, only a small proportion of vaccinated individuals were represented in the national pharmacovigilance system. This low reporting frequency should not be interpreted as indicating that the remaining vaccinees experienced no post-vaccination reactions, because spontaneous surveillance systems are expected to substantially underreport transient and non-serious events. Among reported cases, men had higher overall and serious AEFI reporting rates than women. Higher AEFI reporting rates were also observed among adults aged ≥60 years, particularly in the 80–84-year age group. The most frequently reported symptom in older adults was headache, followed by malaise and fever. Similarly, studies conducted in other countries in the region show more adverse effects in men and those over 60 years of age [35].

Sex-related biological, behavioral, and reporting differences may influence the observed distribution of AEFIs; however, the mechanisms underlying the higher reporting rates among men in our cohort cannot be established from the available pharmacovigilance data [36,37,38]. Beyond safety, advanced age may also influence the immunogenicity of yellow fever vaccination. Immunosenescence can delay the development of neutralizing antibody responses after primary vaccination, although available studies indicate that most healthy older adults ultimately achieve seroprotection and may maintain long-term immunity. Sex-related differences in vaccine-induced immunity have also been described; however, findings specifically for yellow fever vaccination are heterogeneous, and the relationship between sex-specific immune responses and AEFI occurrence remains uncertain. Because the present study did not measure immunogenicity, the higher AEFI reporting rates observed in men cannot be attributed to differences in vaccine-induced immune responses.

Some reports estimate a three- to five-fold increased risk of serious vaccine-associated adverse events in older adults and an increased risk of YEL-AVD among older vaccine recipients [30,31,39,40,41]. Immunosenescence in older adults is considered the biological mechanism that favors replication of the attenuated virus in visceral organs [17,30,31]. Long-term post-vaccination safety assessments and case definitions of viscerotropic disease secondary to vaccination support these age-dependent risk findings [34,39,40,41].

A particularly important finding was the identification of four reports initially coded as viscerotropic syndromes or suspected YEL-AVD among adults aged ≥60 years. The intervals from vaccination to onset of the reported viscerotropic illness were less than 10 days. Two of these reports had fatal outcomes, and after expert causality assessment using the Brighton Collaboration case definition and the national causality-assessment framework, both were classified as coincidental and were not considered vaccine-attributable YEL-AVD. Thus, although all four reported viscerotropic presentations occurred in older adults, pharmacovigilance coding of a clinically compatible syndrome should not be interpreted as confirmation of vaccine-associated YEL-AVD. Of the reports with completed expert assessment, none fulfilled the combined clinical and causality criteria for confirmed vaccine-attributable YEL-AVD. These observations are consistent with the recognized age-related safety concern surrounding YEL-AVD, but given the very small number of reports and incomplete causality information, they should not be interpreted as an estimate of age-specific causal incidence.

Emerging evidence also suggests that impaired type I interferon immunity may predispose some individuals to rare, life-threatening adverse reactions following live-attenuated yellow fever vaccination. Patients with YEL-AVD and YEL-AND have been found to have neutralizing autoantibodies against type I interferons, as well as inherited defects affecting type I interferon signaling. More recently, we also detected anti-type I interferon autoantibodies in a subset of patients with vaccine-associated neurotropic or viscerotropic disease. However, our surveillance dataset did not include biological specimens or immunological testing; therefore, the presence of anti-type I interferon autoantibodies or other defects in interferon-mediated immunity could not be evaluated as potential host susceptibility factors in the present study [42,43].

These findings underscore the importance of defining vaccination recommendations for individuals over 60 years of age. Consequently, it is essential to conduct the individualized risk-benefit assessment recommended by health authorities, such as the Strategic Advisory Group of Experts (SAGE), for people over 60, taking into account the actual risk of exposure [25,28,30]. Similarly, active pharmacovigilance of serious adverse events following immunization should be strengthened, particularly after the first doses, when the risk of viscerotropic disease is highest [28,30].

Regarding symptoms, the literature shows that fever is the most frequent initial symptom (appearing within the first 10 days) in viscerotropic disease. Therefore, our results reinforce the need for early clinical evaluation in cases of fever among vaccinated older adults [34].

Despite evidence of the risk of serious vaccine-associated adverse events, a higher risk of death from yellow fever virus-induced disease has been demonstrated [5]. Vaccine-attributable mortality is therefore a quantitatively smaller event, and overstating it could potentially encourage vaccine hesitancy.

The global Eliminate Yellow Fever Epidemics (EYE) strategy and the most recent WHO guidelines prioritize immunization in at-risk populations, seeking to close gaps in access to vaccination [27,44]. The recent increase in yellow fever cases among unvaccinated populations in Colombia and the region [3,10,13] demonstrates persistent barriers to vaccination and a perception of the vaccine’s lack of safety [26,27,44,45].

In settings of active transmission, the substantial morbidity and mortality associated with yellow fever should be weighed against the low absolute reporting frequency of serious post-vaccination events when conducting individualized benefit–risk assessments in older adults.

## 5. Limitations

This study has several limitations inherent in using secondary pharmacovigilance and vaccination data. First, because AEFI ascertainment relied on spontaneous reporting to VigiFlow, under-reporting is likely, particularly for non-serious, transient, and self-limited events that may not prompt medical consultation or formal notification. Serious AEFIs are more likely to reach healthcare providers and surveillance systems and may therefore be preferentially reported. The very low number of reports during the initial months of the vaccination campaign, followed by a marked increase in non-serious AEFI reporting as vaccination and surveillance activities intensified, supports the possibility of differential reporting over time. Consequently, the rates presented in this study should be interpreted as pharmacovigilance reporting rates rather than estimates of the true incidence of AEFIs, and the available data cannot quantify the magnitude of under-reporting. More broadly, spontaneous pharmacovigilance systems primarily detect signals rather than estimate the true incidence or causal risk of adverse reactions. Such systems are designed to identify unusual or disproportionate patterns of reported events that may represent potential safety concerns and warrant further investigation. Because only a fraction of AEFIs, particularly non-serious and self-limited events, are reported, the absence or frequency of reports cannot be directly translated into population-level incidence. Conversely, an observed pharmacovigilance signal does not by itself establish that vaccination caused the reported event. Accordingly, the age- and sex-related differences identified in this study should be considered hypothesis-generating safety signals that require confirmation through more robust approaches, including active surveillance, prospective cohort studies, record linkage, and appropriately controlled epidemiological analyses.

From a public health perspective, these limitations support strengthening yellow fever vaccine safety surveillance in Colombia, particularly among adults aged ≥60 years. In high- and very-high-risk municipalities, where vaccination must be maintained because of ongoing yellow fever transmission, passive reporting could be complemented by targeted active surveillance of older vaccine recipients, with structured follow-up during the first 30 days after vaccination and more intensive contact during the first days, when most AEFIs in this study occurred. Integrating or routinely linking EPI vaccination records with pharmacovigilance databases could facilitate identification of recently vaccinated older adults, verification of denominators, and more complete ascertainment of serious events. Additional measures should include standardized training of vaccinators and clinicians to recognize and promptly report serious AEFIs and syndromes compatible with YEL-AVD or YEL-AND, simplified electronic reporting pathways, expedited expert causality assessment of serious cases, and periodic feedback to vaccination sites and territorial health authorities on reporting completeness. Patient-facing communication at the time of vaccination should also explain which symptoms warrant medical evaluation and how to report them. Such policies should strengthen vaccine-safety surveillance without creating additional barriers to vaccination, which remains essential in Colombian settings with active yellow fever transmission and substantial disease-related mortality.

Second, increased awareness and surveillance during the yellow fever emergency, particularly among adults aged ≥60 years, may have influenced reporting and could partially explain the higher observed reporting rates in this group. Third, causality assessment was not available for all serious AEFIs; therefore, reported events should not necessarily be interpreted as vaccine-attributable events. Finally, heterogeneity and possible incompleteness in EPI vaccination records may have affected denominator estimates. In addition, individual-level clinical information in the pharmacovigilance reports was incomplete and not consistently available, including comorbidities, frailty, immunosuppression, concomitant medications, previous yellow fever vaccination, vaccine lot, and other potential determinants of AEFI risk. In particular, the inability to distinguish primary vaccinees from previously vaccinated individuals matters because YEL-AVD and YEL-AND have historically been reported predominantly after primary yellow fever immunization. Although the Colombian campaign targeted susceptible persons and did not routinely recommend revaccination when a previous dose could be verified, individuals with undocumented vaccination histories could have been revaccinated. If repeat vaccinees were included in the denominator, the observed reporting rates of these serious vaccine-associated events could be lower than those expected in an exclusively vaccine-naïve population. The available data did not allow for quantification of the proportion of primary versus repeat vaccine recipients. Consequently, the multivariable analysis of factors associated with AEFI seriousness was limited to age and sex, precluding a more granular assessment of independent patient-level risk factors. Residual confounding by unmeasured characteristics, particularly comorbidity and frailty, therefore cannot be excluded when interpreting the observed age- and sex-related differences. These limitations will be further addressed in the prospective phase of the protocol through systematic causality assessment, more complete characterization of comorbidities and other clinical risk factors, and expanded multivariable analyses. Likewise, no biological samples or immunological data were available to assess emerging host susceptibility factors, including neutralizing autoantibodies against type I interferons or inherited defects in type I interferon signaling.

## 6. Conclusions

National pharmacovigilance data showed higher AEFI reporting rates among adults aged ≥60 years than among younger vaccinees, including higher reporting rates of serious events, although the absolute number of reported serious AEFIs remained low. These findings should be interpreted as hypothesis-generating safety signals rather than estimates of causal incidence or vaccine-attributable risk, because spontaneous pharmacovigilance is designed primarily for signal detection and is susceptible to under-reporting, stimulated and differential reporting, and incomplete clinical information. No reviewed fatal outcome was attributed to yellow fever vaccination, and reported viscerotropic clinical presentations should not be considered confirmed YEL-AVD without appropriate causality classification. In settings of active yellow fever transmission, these findings support individualized benefit–risk assessment for older adults together with careful pre-vaccination screening and strengthened pharmacovigilance. In Colombia, this should include targeted active follow-up of older vaccine recipients, linkage between immunization and pharmacovigilance databases, simplified and timely AEFI reporting by healthcare providers and vaccinees, and expedited causality assessment of serious events. These measures should improve safety-signal detection without introducing unnecessary barriers to vaccination in populations at substantial risk of yellow fever. Further studies using active surveillance and more complete individual-level clinical data are needed to quantify vaccine-attributable risks in this population.

## Figures and Tables

**Table 1:** Vaccinated, AEFIs and serious AEFIs per month, Colombia, October 2024–July 2025.

Period	Cases	AEFIs (Mild/Serious)	Vaccinated	Rate per 100,000 Vaccinated (Mild/Serious)
Oct-2024	1	1 (0/1)	126,909	0.79 (0.00/0.79)
Nov-2024	1	1 (1/0)	246,698	0.41 (0.41/0.00)
Dic-2024	0	0(0/0)	237,807	0(0/0)
Jan-2025	3	3 (2/1)	138,147	2.17 (1.45/0.72)
Feb-2025	15	30 (15/15)	148,250	20.24 (10.12/10.12)
Mar-2025	12	23 (8/15)	170,311	13.50 (4.70/8.81)
Apr-2025	112	224 (180/44)	1,129,442	19.83 (15.94/3.90)
May-2025	49	86 (59/27)	869,716	9.89 (6.78/3.10)
Jun-2025	24	39 (31/8)	470,900	8.28 (6.58/1.70)
Jul-2025	15	15 (14/1)	377,786	3.97 (3.71/0.26)
Not classifiable	1	3 (0/3)	-	-
Total	233	425 (310/115)	3,915,966	10.85 (7.92/2.94)

**Table 2:** Rates of AEFIs and serious AEFIs by sex, Colombia, October 2024–July 2025.

Sex	Cases	N AEFIs	Serious AEFIs (%)	Vaccinated	Total Rate [95%CI]	Serious Rates	Mild Rates
Women	115	192	33 (17.2)	2,155,629	8.91 [7.7–10.2]	1.53	7.38
Men	118	233	82 (35.2)	1,761,145	13.23 [11.6–14.9]	4.66	8.57

**Table 3:** Rates of AEFIs and serious AEFIs by broad age group, Colombia, October 2024–July 2025.

Age Group	Absolute Frequency			Vaccinated	Rates × 100,000 Vaccinated	
	Cases	N AEFIs	Serious (%)		Total Rate [95%CI]	Serious Rates [95%CI]
2–19 years	20	33	13 (39.4)	683,038	4.83 [3.2–6.6]	1.90 [0.9–3.1]
20–59 years	82	173	53 (30.6)	2,551,134	6.78 [5.8–7.8]	2.08 [1.5–2.7]
9–23 months	9	10	3 (30.0)	295,138	3.39 [1.4–5.8]	1.02 [0.0–2.4]
≥60 years	122	209	46 (22.0)	386,656	54.05 [46.8–61.5]	11.90 [8.5–15.5]
Total	233	425	115 (27.1)	3,915,966	10.85	2.94

**Table 4:** Binary logistic regression with age and sex as predictors of serious AEFIs.

Variable	OR	95%CI	*p* Value
**Age (years)**	0.991	0.981–1.000	0.058
**Sex (male)**	2.589	1.642–4.155	0.0001

**Table 5:** Clinical characteristics and causality classification of fatal AEFI cases, Colombia, October 2024–July 2025.

Vaccine/Lot	Event	Comorbidities	Age	Causality Classification
Bio-Manguinhos 23K0929	2 reported viscerotropic syndromes, * 1 endocarditis	Hypertension, tricuspid regurgitation, dyslipidemia, osteoarthritis	71–75–76	Coincidental **
Bio-Manguinhos 24F0617	2 sepsis, 1 stroke	Hypertension, chronic gastropathy, COPD, diabetes, Alzheimer’s	81–83–91	Coincident **/Not reported
Bio-Manguinhos 24F0618	Acute Myocardial Infarction	Acute Myocardial Infarction	85	Coincidental **
Lot 23K0929 (no name)	Acute Myocardial Infarction	Hypertension, dyslipidemia, smoking, chest pain	60	Coincidental **
Sanofi 24600832127	Difficulty breathing	No.	75	No.

* Clinical presentation reported as viscerotropic; these cases were classified as coincidental after causality assessment and should not be interpreted as confirmed YEL-AVD. ** Coincidental: the event occurred after vaccination but, following causality assessment, was considered unrelated to the vaccine because an alternative cause or underlying condition provided a more plausible explanation.

## Data Availability

The data presented in this study are available upon request from the corresponding author.
